# Everolimus and long acting octreotide as a volume reducing treatment of polycystic livers (ELATE): study protocol for a randomized controlled trial

**DOI:** 10.1186/1745-6215-12-246

**Published:** 2011-11-21

**Authors:** Melissa Chrispijn, Joost PH Drenth

**Affiliations:** 1 Department of Gastroenterology and Hepatology, Radboud University Nijmegen Medical Centre, Nijmegen, the Netherlands

## Abstract

**Background:**

Polycystic liver disease (PLD) is defined as having more than 20 liver cysts and can present as a severe and disabling condition. Most symptoms are caused by the mass effect of the liver size and include abdominal pain and distension. The somatostatin analogues octreotide and lanreotide have proven to reduce polycystic liver volume. mTOR inhibitors such as everolimus inhibit cell proliferation and might thereby reduce growth of liver cysts. This trial aims to assess the benefit of combination therapy of everolimus and octreotide compared to octreotide monotherapy. In this study we present the structure of the trial and the characteristics of the included patients.

**Methods/design:**

This is a randomized open-label clinical trial comparing the effect of 12 months of everolimus and octreotide to octreotide monotherapy in PLD patients. Primary outcome is change in liver volume determined by CT-volumetry. Secondary outcomes are changes in abdominal symptoms and quality of life. Moreover, safety and tolerability of the drugs will be assessed.

**Discussion:**

This trial will compare the relative efficacy of combination therapy with octreotide and everolimus to octreotide monotherapy. Since they apply to different pathways of cystogenesis we expect that combining octreotide and everolimus will result in a cumulative reduction of polycystic liver volume.

**Trial registration number:**

ClinicalTrials.gov: NCT01157858

## Background

Polycystic liver disease (PLD) is a condition that is arbitrarily defined as the presence of at least 20 liver cysts. The two genetic distinct disorders autosomal dominant polycystic liver disease (PCLD) and autosomal dominant polycystic kidney disease (ADPKD) are most frequently responsible for PLD[1]. In ADPKD there are polycystic kidneys which can be accompanied by liver cysts in up to 87% of the patients, depending on age and severity of renal impairment[2]. The phenotype of PLD can vary widely but in most advanced cases impairs quality of life by the mechanical complaints caused by the massively enlarged liver[3].

Until recently, surgical procedures were the only treatment options to reduce polycystic liver volume. However, these invasive procedures are accompanied by relatively high morbidity and there is a small but detectable procedure related mortality[1]. In the last few years, medical treatment options have been explored as an alternative to invasive therapy. Somatostatin analogues by virtue of reducing cAMP in the cholangiocytes that line the cyst. The first signal of efficacy came from experimental studies in rodent PLD models where octreotide suppressed liver (-19%) and liver cyst (-40%) volumes[4]. This concept was further reinforced by several randomized clinical trials that demonstrated that a 6-12 month somatostatin analogue therapy with octreotide or lanreotide reduces liver volume by 15-38%[5-9]. Recently, mTOR inhibitors have proven to have an effect on polycystic liver volume as well[10].

mTOR inhibitors are immunosuppressants that target and inhibit mTOR, and thereby exert antiproliferative, antiangiogenetic and tumor-progression blocking capabilities that might serve preventing uncontrolled cholangiocyte cell proliferation. Treatment with mTOR inhibitors dramatically reduced cyst volume in experimental models[11-15]. An observational trial in ADPKD patients who received a kidney transplant observed that sirolimus reduced polycystic liver volumes by 12% compared to an increase of 14% in patients who received standard treatment with tacrolimus[10].

We were interested whether mTOR inhibitors would be able to augment the PLD reducing effect of somatostatin analogues. Therefore we set out to design a clinical trial that investigates the effect of combining a somatostatin analogue (octreotide) and a mTOR inhibitor (everolimus) in a randomized fashion to assess whether combination therapy has additional effect over somatostatin analogue monotherapy in PLD volume reduction.

## Patients and Methods

### Study population

All symptomatic PLD patients (≥ 20 liver cysts on CT scanning) with PCLD or ADPKD, that meet the following eligible criteria are suitable for participation in this study.

#### Inclusion criteria

• 18 < age ≤ 70 years

• Polycystic liver disease (PCLD or ADPKD), defined as ≥ 20 liver cysts

• Total liver volume must be at least 2500 mL

• Symptomatic defined as ECOG-PS ≥ 1 (ECOG-Performance Scale: indicates how disease affects the daily living abilities of the patient; scale ranges from 0 to 5 in order of severity), and having at least three out of ten PLD symptoms:

- Abdominal pain

- Abdominal distension

- Abdominal fullness

- Dyspnea

- Early satiety

- Back pain

- Nausea/vomiting

- Anorexia

- Weight loss

- Jaundice

• Informed consent, patients are willing and able to comply with the study drug regimen and all other study requirements

#### Exclusion criteria

• Use of oral contraconceptives or estrogen supplementation

• Females who are pregnant or breast-feeding or patients of reproductive potential not employing an effective method of birth control; women of childbearing potential must have a negative serum pregnancy test within 48 hours prior to the administration of study medication

• Intervention (aspiration or surgical intervention) within three months before baseline

• Treatment with somatostatin analogues within three months before baseline

• Patients with a kidney transplant

• History or other evidence of chronic pulmonary disease associated with functional limitation

• History of severe cardiac disease (e.g. NYHA Functional Class III or IV, myocardial infarction within 6 months, ventricular tachyarrhythmias requiring ongoing treatment, unstable angina or other significant cardiovascular diseases); in addition, patients with documented or presumed coronary artery disease or cerebrovascular disease should not be enrolled

• History or other evidence of severe illness or any other conditions which would make the patient, in the opinion of the investigator, unsuitable for the study

• Symptomatic gallstones (octreotide decreases gall bladder volume)

• Hypercholesterolemia (fasting cholesterol > 8 mmol/l) or hypertriglyceridaemia (> 5 mmol/l) not controlled by lipid lowering therapy

• Granulocytopenia (white blood cell < 3,000/mm3) or thrombocytopenia (platelets < 100,000/mm3)

• Infection with hepatitis B, hepatitis C, HIV, TBC (in medical history)

• Mental illness that interferes with the patient ability to comply with the protocol

• Drug or alcohol abuse within one year of baseline

• Co-medication with strong inhibitor of CYP3A4 and/or P glycoprotein like voriconazole, ketoconazole, diltiazem, verapamil, erythromycin or with a strong CYP3A4 and or P-glycoprotein inductor like rifampicin

• Known hypersensitivity to everolimus or one of its excipients

• Enrolment in another clinical trial of an investigational agent while participating in this study

• Moderate or severe reaction on contrast in medical history

• Treatment with I131 during the course of the trial

• Use of metformine

• Morbus Kahler or Morbus Waldenstrom with excretion of light chains in urine in medical history

• Kidney dysfunction (MDRD-GFR < 60 ml/min/1.73m2 and endogenous creatinine clearance < 60 ml/min, calculated by the Cockcroft-Gault formula); in case of decreased body muscle mass, exact endogenous creatinine clearance is measured using serum and urine creatinine

### Study design and setting

**Figure 1:**
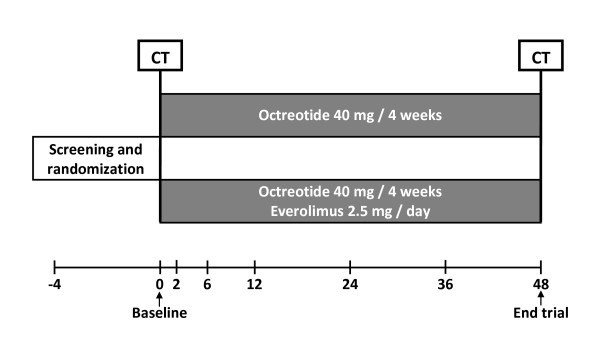
**Trial design of the ELATE trial**. All patients are screened for eligibility and the patients who suit the criteria are randomized in an equal ratio to either the octreotide monotherapy arm or the octreotide and everolimus arm. All patients receive a CT scan at baseline and after 12 months of treatment. Control visits are performed at 2, 6, 12, 24, 36 and 48 after baseline.

The ELATE trial is a single-centre, randomized, open-label parallel study in adult symptomatic PLD due to PCLD and ADPKD. The trial is performed in the Radboud University Nijmegen Medical Centre, Nijmegen, The Netherlands. Duration of treatment is 48 weeks. The design of the trial is showed in Figure 1.

### Randomization, blinding and treatment allocation

Patients are randomized to one of the treatment arms in a 1:1 ratio (Figure 1). An eligible patient is assigned to a trial-code (randomization number). A computer generated randomization list is made by an independent biostatistics unit using a permuted block design with a random block size of 4 to guarantee a balanced allocation. PLD liver volume will be assessed using unmarked CT scans to assure that the assessor is blinded to the assigned treatment. All CT scans will be anonymized and all dates will be removed, so the clinical investigator is unfamiliar which CT scan belongs to which patient and whether the CT scan is dated prior to treatment or after treatment. All CT scans will be assessed by the same operator (MC). The CT scans will be reassessed blindly by an independent investigator.

### Treatment arms

The ELATE trial has two treatment arms. One arm receives a combination of octreotide and everolimus, while the other arm will receive octreotide monotherapy without placebo. Patients in both arms are treated with octreotide LAR (Sandostatine LAR, Novartis) 40 mg intramuscularly every 28 days. Since Sandostatine LAR is not provided in dosages of 40 mg, all patients receive two subsequent injections of 20 mg. Everolimus is started in an initial dose of 2,5 mg daily and is monitored by measuring trough levels. We maintain trough levels within 3-8 ng/mL. Trough levels are assessed two weeks after starting everolimus and a check is performed after 24 weeks, at control visit 4. When trough levels exceed these values, the frequency of dosing is adjusted. Patients were requested to take their medication at fixed times to avoid fluctuations in the trough levels. Furthermore, patients are advised to avoid sun exposure, and avoid drugs or food that are known to interact with everolimus.

### Hypothesis

The main hypothesis of the study is that the combination of everolimus and octreotide gives more reduction of PLD volume when compared to octreotide monotherapy.

### Primary outcome

Primary outcome is the change of total liver volume between the two treatment arms in terms of percentage from baseline to 12 months as determined by CT-volumetry.

### Secondary outcomes

Secondary outcomes of the ELATE trial are change of kidney volume from baseline to 12 months in ADPKD patients as determined by CT-volumetry, change in symptoms, as assessed by GI questionnaire[16], and change in quality of life, as assessed by Euro-QoL questionnaire[17]. Other secondary outcomes are the proportion of patients having any reduction in total liver volume after 12 months and all adverse events in these 12 months. Furthermore, vital signs, laboratory and safety parameters are monitored at all visits during 12 months of treatment.

### Data collection

Patients are seen at baseline, and after 2, 6, 12, 24, 36 and 48 weeks of treatment. During these visits medical history is taken and vital signs and laboratory tests are assessed. During medical history taking patients are asked for concomitant medication and adverse events. A CT scan is made at baseline and after 48 weeks of treatment. In addition we administer GI and EuroQoL questionnaires at these time points. All data are completed on a Case Report Form (CRF) and collected in a database. The parameters that are asked for at the different visits are found below.

#### Baseline

• Written informed consent

• Eligibility criteria check

• Assessment of ECOG-PS score (see *Inclusion criteria*)

• Verification of the diagnosis of polycystic liver disease

• General characteristics: initials, date of birth (dd/mm/yyyy), age (years), gender, weight (kg), height (cm), blood pressure (mmHg), heart rate and ethnic background

• Pregnancy test in females between 18-50 years (<7 days before baseline)

• Hepatitis B, C, HIV, TBC screening: HBsAg, anti-HCV and HIV in blood and Mantoux and chest X-ray

• Serum storage for future reference (6 ml)

• GI symptom questionnaire

• EuroQoL questionnaire

• CT scan of the liver

#### Every visit

• Concomitant medication

• Adverse events

• Drug accountability

• Physical examination, blood pressure and heart rate

• Weight

• Lab hematology

• Lab chemistry

#### End of treatment

• GI symptom questionnaire

• EuroQoL questionnaire

• CT scan with contrast of the liver

### Withdrawal of individual subjects

Patients must be withdrawn from the study for any of the following reasons:

•Withdrawal of informed consent

•Pregnancy

•Consistently fails to adhere to study drug regimen or protocol requirements

•Study drug discontinuation

•Unacceptable toxicity (grade 4 adverse events)

•Surgical intervention during trial

•Patients also should be withdrawn at any time if the investigator concludes that it would be in the patient's best interest for any reason.

If premature withdrawal occurs for any reason, the investigator must determine the primary reason for a patient's premature withdrawal from the study and record this information on the Study Completion CRF (case report form). We will not allow replacement of study participants into the study.

### Study procedures

**Figure 2:**
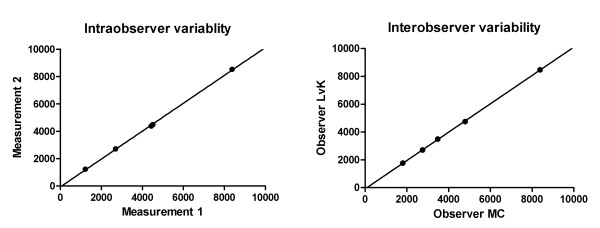
**Intraobserver and interobserver variability**. The figure on the **left panel **shows the intraobserver variability, which is the accuracy of a single researcher that has measured the same CT scan at two different times. The correlation between the consequent measurements is 1.00 (p < 0.01). The figure on the **right panel **shows the interobserver variability, which is the accuracy between two researchers who measured the same CT scan. The correlation between the measurements is 1.00 (p < 0.01).

All patients will be subjected to an abdominal CT with lower radiation dose than usual, and with intravenous contrast, following a standard protocol designed for this trial. CT scans will be anonymized as to allow a blinded assessment of the liver volume. The liver volume will be measured using sequential measurement of the liver outline of subsequent slices[7]. The slices will be outlined manually every 9-10 mm from the cranial to caudal until the total liver has been captured. We use Pinnacle3® v8.0d (Philips Electronics NV, Eindhoven, the Netherlands). This software interpolates the intermediate slices and calculated the areas within the indicated circumference. The intraobserver variability has been assessed by correlating 2 measurements of 5 different CT scans by the first author (MC). Subsequently, interobserver variability was assessed by correlating 2 measurements that were done by 2 independent researchers of 5 different CT scans. The results of these assessments are shown in Figure 2.

### Sample size calculation

Somatostatin analogues reduce liver volume by 57 mL after 12 months of treatment[7], while mTOR inhibitors give a reduction of liver volume of 223 mL[10]. We assumed that the common standard deviation is 130 mL using a two group t-test with a 0.01 two-sided significance level. To detect a difference in means of 166 mL with 90% power, a sample size of 20 in each group is needed. Taken into account a dropout rate of 10%, the minimal sample size needs to be 22 patients per group, so the group totals 45 patients.

### Statistical analysis

Analyses will be performed using SPSS software. Intention-To-Treat analyses will be used for all clinical outcome variables. The Intention-To-Treat population includes all patients who received at least one dose of study medication. When patients have prematurely terminated the trial, they are asked to undergo a CT scan after stop of treatment. These values will be used instead of end-of-treatment values for ITT-analysis. When no CT scan is available after premature termination of the trial or the patients drops out in the first month of the trial, the value at start of treatment will be used for ITT-analysis. We use "last value carried forward" for missing observations other than CT scan. We will provides a sensitivity analysis for missing data assumptions. Parallel analyses conducted on the Per-Protocol population will be performed. The Per-Protocol population is defined as all patients who have received all 12 injections of octreotide within a week around the set date, and in the combination therapy group, all patients who have received at least 80% of the total amount of dosages of everolimus at one year from baseline.

Disease progression will be determined primarily by dividing the change between the baseline and final scans by the duration of follow-up. The volume of the liver will be determined as indicated before.

Treatment comparisons for the continuous outcome variables will be based on an analysis of covariance (ANCOVA model), with baseline and treatment included as covariates and the absolute change from baseline as the dependent variable.

For the primary endpoint, the effect of treatment will be analyzed using the Mann Whitney U test. All laboratory results out of the normal range will be listed. All statistical analyses will be two-sided with a critical significance level of 5%. Frequency tables will be compiled for Adverse Events classified according to the standard WHO-ART Body System Dictionary and preferred terms.

### Ethics

The protocol and the patient information forms were approved by the Medical Ethics Committee. This study is performed in accordance with the protocol, the guidelines of Good Clinical Practice/ICH, the principles of the Declaration of Helsinki 1964 as modified by the 52nd WMA General Assembly, Edinburgh, Scotland, October 2000 including two notes of clarification paragraph 29 and 30, and the local national laws governing the conduct of clinical research studies.

## Discussion

The study has been submitted to clinicaltrials.gov and the trial identifier is NCT01157858. The ELATE trial is designed to determine whether combination therapy of everolimus and octreotide is more effective in reducing polycystic liver volume in PLD patients than octreotide monotherapy. Furthermore, we will assess changes in abdominal symptoms and quality of life.

**Figure 3:**
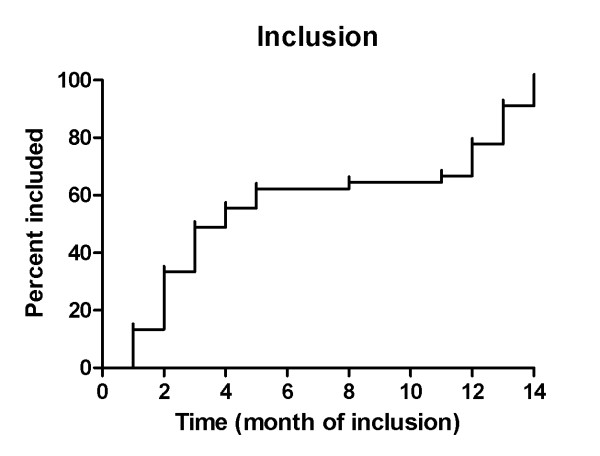
**Course of inclusion**. This figure shows at which frequency the patients entered the trial. In the first 5 months of inclusion was very rapid, but then went flat for a few months. At month 11 the protocol was changed to such an extent that ADPKD patients were also allowed to participate in the trial. This reignited inclusion and the intended sample size was reached within 3 months.

All patients were included between June 2010 and July 2011 and the last patient will complete the trial in July 2012. Initially, we intended to include only PCLD patients, because we wanted to keep the group as unmixed as possible. However, after a rapid inclusion in the first months, inclusion slowed because of an insufficient number of patients who met the inclusion criteria (Figure 3). The prevalence of PCLD is only 1:158.000 [3], while that of ADPKD, another disorder with PLD as a presenting feature, has been estimated at 1:1000 [18]. After reconsideration and in an effort to provide adequate power to the study the inclusion criteria were expanded and ADPKD patients were also allowed to participate. Finally, 45 patients were randomized to either of the treatment arms. Baseline characteristics of all trial participants are stated in Table 1. The groups were equal, as there were no differences found between the treatment arms.

**Table 1:** Baseline characteristics of patients in the ELATE trial

	Octreotide/everolimus (n = 22)	Octreotide (n = 23)
**Age (yr)**	52.9 (8.7)	50.7 (8.0)
**Gender (m/f)**	3/19	2/21
**Diagnosis (PCLD/ADPKD)**	9/13	6/17
**Mutation in PCLD patients**		
***PRKCSH***	10	5
***SEC63***	1	4
**Body mass index (kg/m^2^)**	28.0 (3.5)	28.3 (3.9)
**Systolic blood pressure (mmHg)**	134 (14)	135 (15)
**Diastolic blood pressure (mmHg)**	87 (11)	84 (9)
**Haemoglobin (mmol/L)**	8.2 (0.7)	8.2 (0.7)
White blood count (*10^9^/L)	6.5 (1.6)	6.2 (1.4)
Thrombocyte count (*10^9^/L)	212 (56)	210 (62)
**Creatinine (μmol/L)**	72 (16)	68 (11)
**Albumin (g/L)**	40 (3)	40 (2)
**Bilirubin (μmol/L)**	14 (7)	14 (5)
**γ-Glutamyl Transferase (U/L)**	162 (131)	154 (122)
**Glucose (mmol/L)**	5.0 (0.5)	5.0 (0.8)
**Cholesterol (mmol/L)**	4.9 (0.9)	4.7 (1.0)
**CA19-9 (E/mL)**	123 (168)	134 (172)

All continuous variables are stated as mean (SD)

All PCLD patients underwent mutation analysis, no mutation analysis was performed on ADPKD patients

There were no differences between the treatment arms

There is a fair amount of data from a number of clinical trials that demonstrated the efficacy of somatostatin analogues on PLD volume[5-9,19]. The effect size however differs widely. On the basis of clinical case observations we reported that somatostatin analogues reduced PLD volume by 14-38%[5]. In a subsequent case series of 8 patients we found a reduction of 3.0%[6]. A formal 6-month RCT in 54 patients demonstrated that lanreotide reduced PLD volume by 2.9% when compared to baseline values[7]. Two other RCTs showed that octreotide LAR 40 mg reduced liver volumes by 4.9% when given for 12 months[8], while 6 months octreotide 40 mg decreased total liver volume by 4.4%[9].

Therefore it is reasonable to assume that we will see a decrease of PLD volume during the course of our trial. The key question is whether an add-on treatment with everolimus will be able to further decrease PLD volume to values seen in the observational study by Qian[10]. Just prior to start of recruitment of this trial the results from 2 landmark papers studying the effect of mTOR inhibitors in ADPKD were published[20,21]. The SUISSE ADPKD group performed a 18-month randomized open-label clinical trial with sirolimus 2 mg compared to standard treatment. After 18 months of treatment, there was no difference in kidney cyst growth between the groups[20]. Another group studied everolimus in 433 ADPKD patients in a 2-year, double-blind trial. In contrast to the SUISSE trial, everolimus attenuated the increase of total kidney volume, but did not slow the progression of renal impairment[21]. PLD liver volume was not assessed in these trials. The most important difference with our study is that we use PLD volume reduction as the primary outcome, while these 2 trials used a renal endpoint as a primary outcome measure.

PLD liver volume was assessed through a retrospective analysis of a trial that compared a sirolimus-containing immunosuppression regimen with a tacrolimus-containing regimen in 16 renal transplant recipients with ADPKD. Sirolimus given for an average of 19 months decreased PLD volume by 11.9%, whereas tacrolimus for a comparable duration increased PLD volume by 14.1%[10]. These data led to the assumption that a large effect size of everolimus treatment is realistic.

We have intentionally chosen to combine octreotide and everolimus instead of introducing an everolimus monotherapy arm, because we expect to find a larger reduction of liver volume with combination therapy than with monotherapy. We hope to detect an additional effect, because combination treatment targets two divergent pathways of cystogenesis. Above all, the structure of our trial addresses the issue whether add-on therapy augments the established effect of octreotide.

As with the 2 other trials employing mTOR inhibitors in ADPKD[20,21] we elected not to use a placebo in the control arm. The therapeutic drug monitoring of everolimus effectively unblinds the trial. In addition, we expect that patients will have significantly more side-effects resulting from everolimus treatment than from octreotide. Patient will recognise this and therefore blinding will be ineffective. However, we will measure PLD volume blind to date or patient, since all CT scans will be randomized and disposed of any study dates. This assures that the assessor is unaware whether the CT scan was made at baseline or on follow-up.

Everolimus is initially dosed at 2.5 mg daily and trough levels are maintained between 3-8 ng/mL to have as minimal adverse events as possible. We have adapted our everolimus treatment regimen following those used in solid organ transplantation. Here, dosages are relatively well tolerated. In addition, the treatment schedule used in our RCT is equivalent to the dosages used in the previous trials using mTOR inhibitors in ADPKD patients[20,21].

We believe that there is still an ongoing quest for drugs that can further decrease PLD liver volume and thereby improve quality of life. Consequently, we want to assess whether the combination of somatostatin analogues and mTOR inhibitors works cumulative on reduction of liver volume. Furthermore, by exploring all possible medical treatment options, we eventually hope to contribute a treatment protocol to the field that most effectively reduces symptoms and signs of PLD.

## Trial status

Ongoing

## Abbreviations

PLD: polycystic liver disease, ADPKD: autosomal dominant polycystic kidney disease, PCLD: isolated polycystic liver disease, mTOR: mammalian target of rapamycin, CT: computerized tomography, PCK: polycystic kidney

## Competing interests

This study was funded by Novartis. The sponsor participated in initial discussions regarding study design and protocol development. The sponsor had no role in the conduct of the study; the collection, management, analysis, or interpretation of data; or in the preparation, review, or approval of the manuscript.

## Authors' contributions

MC participated in the design and coordination of the study, required the data and performed the statistical analysis. JD conceived of the study, and participated in its design. MC and JD have been involved in drafting the manuscript and revising it critically for important intellectual content. Both authors read and approved the final manuscript.
